# Surface‐Based Morphometry Analysis of the Cerebral Cortex in Patients With Probable Idiopathic Rapid Eye Movement Sleep Behavior Disorder

**DOI:** 10.1002/brb3.70057

**Published:** 2024-09-30

**Authors:** Milad Najafzadeh, Athareh Saeeidian‐Mehr, Hossein Akbari‐Lalimi, Zohre Ganji, Shahrokh Nasseri, Hoda Zare, Luigi Ferini‐Strambi

**Affiliations:** ^1^ Department of Medical Physics, Faculty of Medicine Mashhad University of Medical Sciences Mashhad Iran; ^2^ Department of Radiology, Faculty of Para‐Medicine Hormozgan University of Medical Sciences Bandar Abbas Iran; ^3^ Medical Physics Research Center Mashhad University of Medical Sciences Mashhad Iran; ^4^ Vita‐Salute San Raffaele University Milan Italy; ^5^ Division of Neuroscience, Sleep Disorders Center San Raffaele Scientific Institute Milan Italy

**Keywords:** cortical analysis, neurodegenerative diseases, probable idiopathic rapid eye movement sleep behavior disorder (iRBD)

## Abstract

**Introduction:**

Strong indications support the notion that idiopathic rapid eye movement (REM) sleep behavior disorder (iRBD) acts as a precursor to multiple α‐synucleinopathies, including Parkinson's disease and dementia with Lewy bodies. Despite numerous investigations into the alterations in cortical thickness and the volume of subcortical areas associated with this condition, comprehensive studies on the cortical surface morphology, focusing on gyrification and sulcal depth changes, are scarce. The purpose of this research was to explore the cortical surface morphology in individuals with probable iRBD (piRBD), to pinpoint early‐phase diagnostic markers.

**Methods:**

This study included 30 piRBD patients confirmed using the RBD Screening Questionnaire (RBDSQ) and 33 control individuals selected from the Parkinson's Progression Markers Initiative (PPMI) database. They underwent neurophysiological tests and MRI scans. The FreeSurfer software was utilized to estimate cortical thickness (CTH), cortical and subcortical volumetry, local gyrification index (LGI), and sulcus depth (SD). Subsequently, these parameters were compared between the two groups. Additionally, linear correlation analysis was employed to estimate the relationship between brain morphological parameters and clinical parameters.

**Results:**

Compared to the healthy control (HC), piRBD patients exhibited a significant reduction in CTH, LGI, and cortical volume in the bilateral superior parietal, lateral occipital, orbitofrontal, temporo‐occipital, bilateral rostral middle frontal, inferior parietal, and precentral brain regions. Moreover, a significant and notable correlation was observed between CTH and Geriatric Depression Scale (GDS), letter–number sequencing (LTNS), the Benton Judgment of Line Orientation (BJLO) test, and the symbol digit modalities test (SDMT) in several brain regions encompassing the motor cortex.

**Conclusion:**

Patients with piRBD displayed widespread atrophy in various brain regions, predominantly covering the motor and sensory cortex. Furthermore, LGI could serve as a prognostic biomarker of disease's progression in piRBD.

## Graphical abstract

Local Gyrfication index could serve as a prognostic biomarker of disease's progression in piRBD.

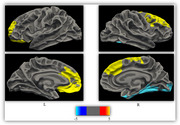

## 1 Introduction

Rapid eye movement (REM) sleep behavior disorder (RBD) falls under the category of REM parasomnias, characterized by unwanted physical activities during sleep, such as laughing, speaking, yelling, and significant movements of the body and extremities (Miglis et al. 2021; Dauvilliers et al. 2018). Polysomnography, complemented by video and audio monitoring, stands as the definitive method for diagnosing RBD while ruling out other conditions with overlapping symptoms like obstructive sleep apnea, night‐time hallucinations, and confusional awakenings (Sateia 2014). Recognized increasingly as a precursor to several neurodegenerative disorders, including Parkinson's disease (PD), multiple system atrophy (MSA), and dementia with Lewy bodies (DLB), idiopathic RBD accounts for around 60% of RBD instances (Ferini‐Strambi 2011). Research shows that the likelihood of developing these conditions escalates over time, positioning idiopathic RBD (iRBD) as the foremost clinical indicator of such diseases to this point (Gnarra et al. 2023). This underscores the urgency in pinpointing cerebral biomarkers in individuals with iRBD to deepen our understanding of its origins, assist healthcare professionals in tracking disease progression, and facilitate prompt intervention. With iRBD being identified as the most accurate and specific harbinger of neurodegenerative diseases, there is an intensified focus on delineating cerebral biomarkers indicative of neurodegeneration within this condition (Miglis et al. 2021).

The genesis of RBD could be linked to impairments within the lower regions of the brainstem nuclei, the striatum, substantia nigra, and the limbic system, suggesting a broad neural disturbance (Campabadal et al. 2021). Studies analyzing the volumetric morphometry in individuals with iRBD have documented significant degeneration in the cerebral gray matter (GM) areas, notably the external and internal segments of the pallidum, the cerebellum, postcentral gyrus, cingulate gyrus, precuneus, and the Rolandic operculum, all primarily due to functional disruptions (Campabadal et al. 2021). Research employing voxel‐based morphometry (VBM) unveiled volumetric diminishments in the layers of the frontal, temporal, parietal, and occipital cortices (Chen et al. 2022), with an increase in the volume of deep brain nuclei as a possible compensatory process. Surface‐based morphometry analyses identified a decrease in cortical thickness (CTH) in several brain regions, including the postcentral gyrus, fusiform regions, and right superior frontal and lateral occipital areas, which are associated with posterior‐based cognitive dysfunction (Valli et al. 2022). Furthermore, longitudinal studies have shown that over time, more brain regions become involved in CTH reduction (Campabadal et al. 2020; Pereira et al. 2019; Campabadal et al. 2019).

Previous studies on cortical changes in iRBD have based their analysis on brain CTH, VBM, and brain shape analysis (Chen et al. 2022; Campabadal et al. 2019). However, brain CTH can be prone to local measurement noise and does not fully define brain cortical morphology (Dahnke, Yotter, and Gaser 2013; Kharabian Masouleh et al. 2020). In addition to reduced CTH, factors, such as increased age, the depth of brain sulci, and decreased complexity of brain gyri, also contribute to brain atrophy (Erten‐Lyons et al. 2013). Using surface‐based morphometry (SBM), it is possible to calculate not only brain CTH but also sulcus depth (SD) and the local gyrification index (LGI).

The process of cerebral cortex gyrification involves changes in the morphology of the cortex to form folds and grooves (White et al. 2010). The LGI is a measure used to quantify cortical gyrification (Schaer et al. 2008). Sulcus depth is an important biomarker for morphology in neuroscience and neuropsychiatric diseases (Yun et al. 2013). The process of folding and deepening of sulci may be related to functional areas and occurs during brain development, including initial radial growth and subsequent tangential expansion. SD is sensitive to brain atrophy resulting from reduced CTH, decreased gyral folding, and diminished white matter (WM) volume. As brain atrophy progresses, a reduction in gyral volume and an increase in cerebrospinal fluid (CSF) cause the sulci to become shallower. Previous neuroimaging studies have reported alterations in LGI and SD in several brain regions in patients with PD, Alzheimer's disease, major depressive disorder, autism spectrum disorder, multiple sclerosis, Huntington's disease, and schizophrenia compared to healthy controls (HCs) (Zhang et al. 2023; Wang et al. 2021; Núñez et al. 2020; Wu et al. 2021; Long et al. 2020; Li et al. 2021; Maier et al. 2018; Tan et al. 2022; Mangin et al. 2020; Stoebner et al. 2023; Sasabayashi et al. 2020; Wei et al. 2022). Additionally, several longitudinal studies have demonstrated a decrease in CTH in the patient with iRBD (Campabadal et al. 2019; Rahayel et al. 2023). A significant reduction in the right hippocampal volume and subcortical volume was also confirmed in the iRBD group compared to controls (Campabadal et al. 2019). However, there is a lack of studies that concurrently examine CTH, volumes of various brain regions and subcortical areas, LGI, and SD in patients with iRBD, leaving these parameters unknown in comparison to HCs. This study aims to address this research gap from previous studies using a focused, classified approach. A comprehensive examination of cortical features may reveal more cortical changes associated with iRBD. In the current study, we focused on cortical and subcortical levels using complementary features, such as CTH, SD, and LGI, as well as cortical and subcortical volumes, to highlight significant differences in various brain regions.

## 2 Materials and Methods

### 2.1 Participants

The data utilized in this article were taken from the Parkinson's Progression Markers Initiative (PPMI) database (Marek et al. 2011) (http://www.ppmi‐info.org), a longitudinal, multi‐center clinical study of de novo, drug‐naïve patients with PD, whose goal is to identify biomarkers of disease progression. The data used for analysis are openly available. For the purposes of this study, only iRBD patients and HCs who had undergone T1‐ and T2‐weighted scans, passing quality control before and after image preprocessing, were included.

The diagnosis of iRBD was performed based on the clinical history and the RBD Screening Questionnaire (RBDSQ), which is a 10‐item structured questionnaire characterizing dreams and dream enactment behaviors, given that there is currently no polysomnography data in the PPMI database (Stiasny‐Kolster et al. 2007). It has also been confirmed by polysomnography (Stiasny‐Kolster et al. 2007). Hence, participants in this group were described as probable iRBD (piRBD). A cutoff score of ≥ 5 was used for RBD detection (Stiasny‐Kolster et al. 2007). Patients in this group were also free from any neurological diseases.

The HC group was required to have no neurological performance deficits, no first‐degree family members with PD, no cognitive disorders (MOCA ≥ 27), no neurodegenerative disorders (RBDSQ < 5), and no olfactory system dysfunction for their age and sex.

Overall, 30 piRBD patients and 33 individuals in the HC group were enrolled in this study. Informed written consent was obtained from subjects or their representatives at each participating center in the PPMI study.

### 2.2 Clinical Evaluations

Each participant underwent a thorough assessment encompassing motor, non‐motor, and cognitive domains. The severity of motor symptoms and disease progression was gauged utilizing the Movement Disorders Society Unified Parkinson's Disease Rating Scale (MDS‐UPDRS) Part III and the Hoehn and Yahr (H&Y) scale. Non‐motor functions were scrutinized using the University of Pennsylvania Smell Identification Test (UPSIT), the Epworth sleepiness scale (ESS), the RBDSQ for RBD, and the Geriatric Depression Scale 15 (GDS‐15) for depressive symptoms.

Cognitive evaluations comprised the Montreal Cognitive Assessment (MoCA) for overall cognitive function, the Benton Judgment of Line Orientation (BJLO) test for visuospatial abilities, the letter–number sequencing (LTNS) for executive function assessment, and the symbol digit modalities test (SDMT).

### 2.3 MRI Acquisition

MRI T1 scans were conducted using scanners with magnetic field strengths of either 1.5 or 3 T at different facilities, utilizing the MPRAGE imaging protocol. Standard settings for MRI scans comprised a repetition time (TR) ranging from 5 to 11 ms, an echo time (TE) between 2 and 6 ms, a slice thickness of 1.5 mm, no interslice gap, voxel dimensions of 1 × 1 × 1.2 mm^3^, and a matrix size of 256 × 256 px^2^.

### 2.4 Surface‐Based Analysis of MR Data

The acquired images, originally in DICOM format, underwent conversion to NIfTI format via the MRIcroGL tool (https://www.mccauslandcenter.sc.edu/mricrogl/). Following this, measurements of CTH, LGI, and cortical SD were conducted using FreeSurfer software version 7.2 (http://www.surfer.nmr.mgh.harvard.edu/). The process of surface reconstruction has been detailed in prior studies. In brief, image processing included intensity normalization, skull stripping, tessellation of gray/WM boundaries, inflation of the folded surface tessellation pattern, and automatic correction of topological defects. This surface then served as the starting point for a deformable surface algorithm to identify GM, WM, and CSF surfaces. This method uses intensity information and continuity of surfaces in deformation processes to generate CTH. CTH is calculated as the shortest distance from the GM/WM boundary to the GM/CSF interface at each vertex on the separated surface. CTH maps were then smoothed and averaged for all subjects using a filter with a full width at half maximum (FWHM) of 10 mm.

In the next step, LGI was calculated by measuring the ratio of the local surface area to the outer body layer that tightly wraps the pial surface, indicating the brain sulci located at the intended location and reflecting the complexity of cortical folding. In the final stages of analysis, the LGI for both the left and right hemispheres was smoothed using a spherical Gaussian filter with an FWHM of 10 mm to ensure a normal distribution of results. Each LGI value was mapped onto a spherical coordinate system (fsaverage), followed by the application of a spherical Gaussian filter with an FWHM of 5 mm to smooth each mapped image.

For the analysis of the SD, files labeled rh.sulci and lh.sulci were employed. These files provide information about the distance of a specific vertex point on a surface from a hypothetical intermediate surface situated between the sulci and gyri. This intermediate surface is selected such that the “average” of all these displacements is zero. The “sulc” then serves as an indicator of the linear distance and displacements, signifying how “deep” or “elevated” the brain's gyri are.

### 2.5 Statistical Analyses

#### 2.5.1 Clinical Group Comparisons at Baseline

The differences between groups in clinical and demographic variables were analyzed using chi‐squared (*χ* ^2^) and *t*‐tests in the R software package, version 4.3.1 (http://www.r‐project.org), while controlling for age, sex (for motor and non‐motor variables), and education level (for cognitive variables). The Bonferroni method was employed to account for multiple analyses, and statistical significance levels were set at a *p* value of less than 0.05. To evaluate differences in morphometric features (CTH, cortical and subcortical volume, LGI, and SD), a vertex‐wise analysis was conducted between the two groups, adjusting for age, sex, education level, and total intracranial volume (TIV). Results were reported for clusters equal to or larger than 30 mm^3^. A cluster‐wise correction for multiple comparisons was performed using Monte Carlo simulation with 10,000 permutations (cluster‐wise threshold *p* < 0.05), and analyses were conducted separately for each hemisphere. To test both hemispheres, results were adjusted using the Bonferroni method.

#### 2.5.2 Correlation Analysis

A general linear model (GLM) was used to investigate whether there was a correlation between the average morphological surface analyses of the brain and clinical variables. This analysis was performed for the region showing significant difference between two groups. The Bonferroni analysis was applied for multiple comparison corrections, and a *p* value of less than 0.05 was used to identify clusters with significant differences. For clusters that showed a significant relationship among CTH, LGI, cortical volume, and SD and the mentioned variables, average values of the morphological surface analyses of the brain were extracted. Finally, a linear correlation analysis was used to examine the relationship between the morphological surface analyses of the brain and clinical variables, including MDS‐UPDRS III, H&Y, UPSIT, ESS, RBDSQ, and GDS.

## 3 Results

### 3.1 Clinical Differences Among Groups

The characteristics of the study's participants are presented in Table 1, showcasing both demographic and clinical details. The study comprised 31 individuals in the piRBD group and 32 HCs. Initially, the data's normal distribution was evaluated through the Shapiro–Wilk test using R software. Compared to the HC, there was a significant difference in age (Cohen's *d* = −0.63, *p* = 0.012) and sex distribution (Cohen's *d* = −0.3, *p* = 0.016) in the iRBD group. They also had higher MDS‐UPDRS III scores (Cohen's *d* = −0.57, *p* = 0.35) and RBDSQ scores (Cohen's *d* = −3.37, *p* < 0.001), and lower SDMT scores (Cohen's *d* = 1.18, *p* < 0.001) (refer to Table 1).

**TABLE 1:** Clinical characteristics of idiopathic rapid eye movement sleep behavior disorder (iRBD) patients and controls.

Demographic data/clinical	iRBD	HC	iRBD vs. HC (*p* value)	Effect size
Sex (M/F)	22/8	13/20	**0.016**	**−0.3**
Age (mean, SD)	68.06 (6.18)	63.28 (8.63)	**0.012**	**−0.63**
Education (mean, SD)	16.07 (2.73)	16.06 (4.08)	0.99	
MDS‐UPDRS III	3.83 (6.02)	1.3 (2.17)	**0.035**	**−0.58**
RBDSQ	8.37 (2.64)	1.53 (1.26)	**< 0.001**	**−3.37**
GDS	5.2 (1.13)	5.4 (1.02)	0.43	
MoCA	26.83 (2.28)	27.68 (2.13)	0.13	
BJLO	4.77 (5.67)	6.44 (6.74)	0.28	
LTNS	4.83 (5.93)	6 (6.5)	0.45	
SDMT	39.17 (7.29)	49.38 (9.720)	**< 0.001**	**1.18**

*Note*: Data are mean ± standard deviation.

Abbreviations: BJLO, Benton Judgment of Line Orientation; GDS, Geriatric Depression Scale; HC, healthy control; LTNS, letter–number sequencing; MDS‐UPDRS, Movement Disorders Society Unified Parkinson's Disease Rating Scale; MoCA, Montreal Cognitive Assessment; RBDSQ, RBD Screening Questionnaire; SD, sulcus depth; SDMT, symbol digit modalities test.

### 3.2 MRI Differences Among Groups

#### 3.2.1 Cortical Thickness

**FIGURE 1:**
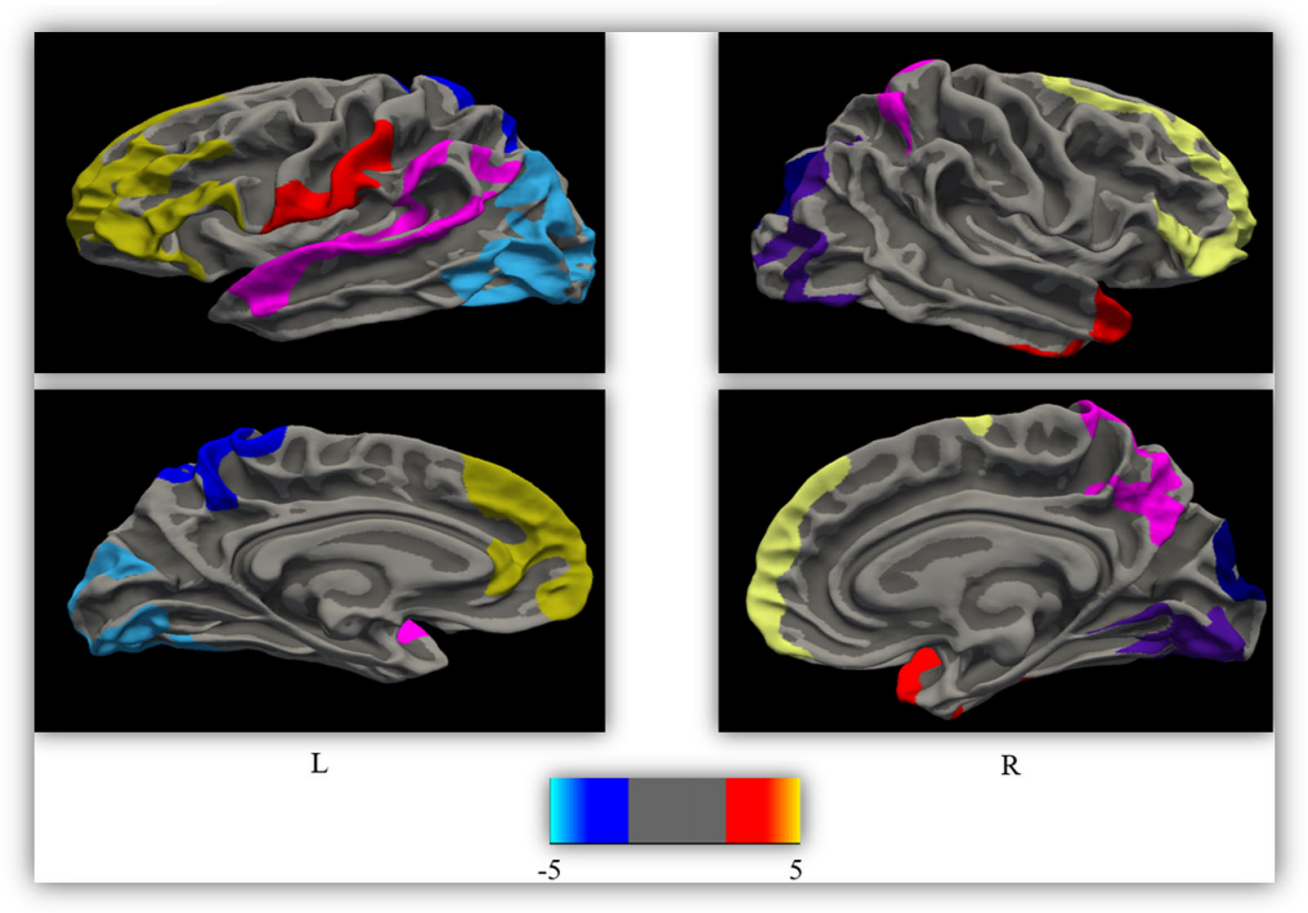
Cortical thinning in patients with iRBD versus HC regions where cortical thickness abnormalities were found in patients with iRBD included right pars orbitalis, right inferior and superior parietal, right cuneus, right temporal pole, left lateral occipital, left superior temporal, left superior parietal, and left precentral. Results of between‐group comparison were performed using a vertex‐wise analysis (Bonferroni‐corrected with Monte Carlo simulation using a *p* value set at < 0.05). The displayed color bar is in the logarithmic scale of *p* values (−log10 [*p* value]). HC, healthy control; iRBD, idiopathic rapid eye movement sleep behavior disorder; L, left hemisphere; R, right hemisphere.

In piRBD patients, a significant reduction in CTH was observed in various brain regions. Specifically, the orbitalis (Cohen's *d* = 2.41, *p* < 0.001), inferior parietal (Cohen's *d* = 2.41, *p* < 0.001), precuneus (Cohen's *d* = 2.41, *p* < 0.001), superior parietal (Cohen's *d* = 2.45, *p* = 0.001), temporal pole (Cohen's *d* = 2.45, *p* = 0.001) on the right side, as well as the G (Cohen's *d* = 2.43, *p* < 0.001), lateral occipital G (Cohen's *d* = 2.43, *p* < 0.001), superior temporal (Cohen's *d* = 2.43, *p* < 0.001), superior parietal (Cohen's *d* = 2.43, *p* < 0.001), and precentral G (Cohen's *d* = 2.43, *p* < 0.001) on the left side, all showed notable thinning (refer to Table 2 and Figure 1).

**TABLE 2:** Regions that showed significant differences in cortical morphometry between idiopathic rapid eye movement sleep behavior disorder (iRBD) patients and healthy control (HC).

Cluster number	Size (mm^3^)	*p* value	Effect size (Cohen's *d*)	*N*_vertices_	*X*_Tal_	*Y*_Tal_	*Z*_Tal_	Anatomical regions
CTH								
1	5573.94	< 0.001	2.41	8570	41.7	39.6	−6.0	R pars orbitalis
2	4907.98	< 0.001	2.41	6862	35.6	−76.2	37.1	R inferior parietal
3	1986.28	< 0.001	2.41	4695	9.4	−59.5	40.8	R precuneus
4	1748.86	0.001	2.45	2349	20.2	−85.5	24.9	R superior parietal
5	1481.25	0.005	2.45	2599	35.7	15.4	−37.0	R temporal pole
6	7926.44	< 0.001	2.43	12429	−37.5	−37.5	−37.5	L rostral middle frontal
7	7758.20	< 0.001	2.43	11335	−43.7	−43.7	−43.7	L lateral occipital
8	4010.93	< 0.001	2.43	8813	−57.6	−57.6	−57.6	L Superior temporal
9	1996.90	< 0.001	2.43	4629	−12.4	−12.4	−12.4	L Superior parietal
10	1835.41	< 0.001	2.43	4318	−56.6	−56.6	−56.6	L precentral
LGI								
1	5327.44	< 0.001	2.2	9179	28.1	−55.5	−14.3	R fusiform
2	5043.64	< 0.001	2.2	9034	28.0	32.8	31.2	R rostral middle frontal
3	5925.94	< 0.001	2.30	10135	−22.4	11.6	−18.7	L lateral orbitofrontal
Cortical volume								
1	1472.77	0.005	2.67	2051	−7.9	58.4	−5.2	L medial orbitofrontal
2	1176.58	0.03	2.45	2743	−13.2	59.7	58.3	L superior parietal

*Note*: L, left; *N*_vertices_, vertex number at maximum; R, right; *X*_Tal_, Talairach coordinates for *X* axis; *Y*_Tal_, Talairach coordinates for *Y* axis; *Z*_Tal_, Talairach coordinates for *Z* axis.

Abbreviations: CTH, cortical thickness; LGI, local gyrification index.

#### 3.2.2 Local Gyrification Index

**FIGURE 2:**
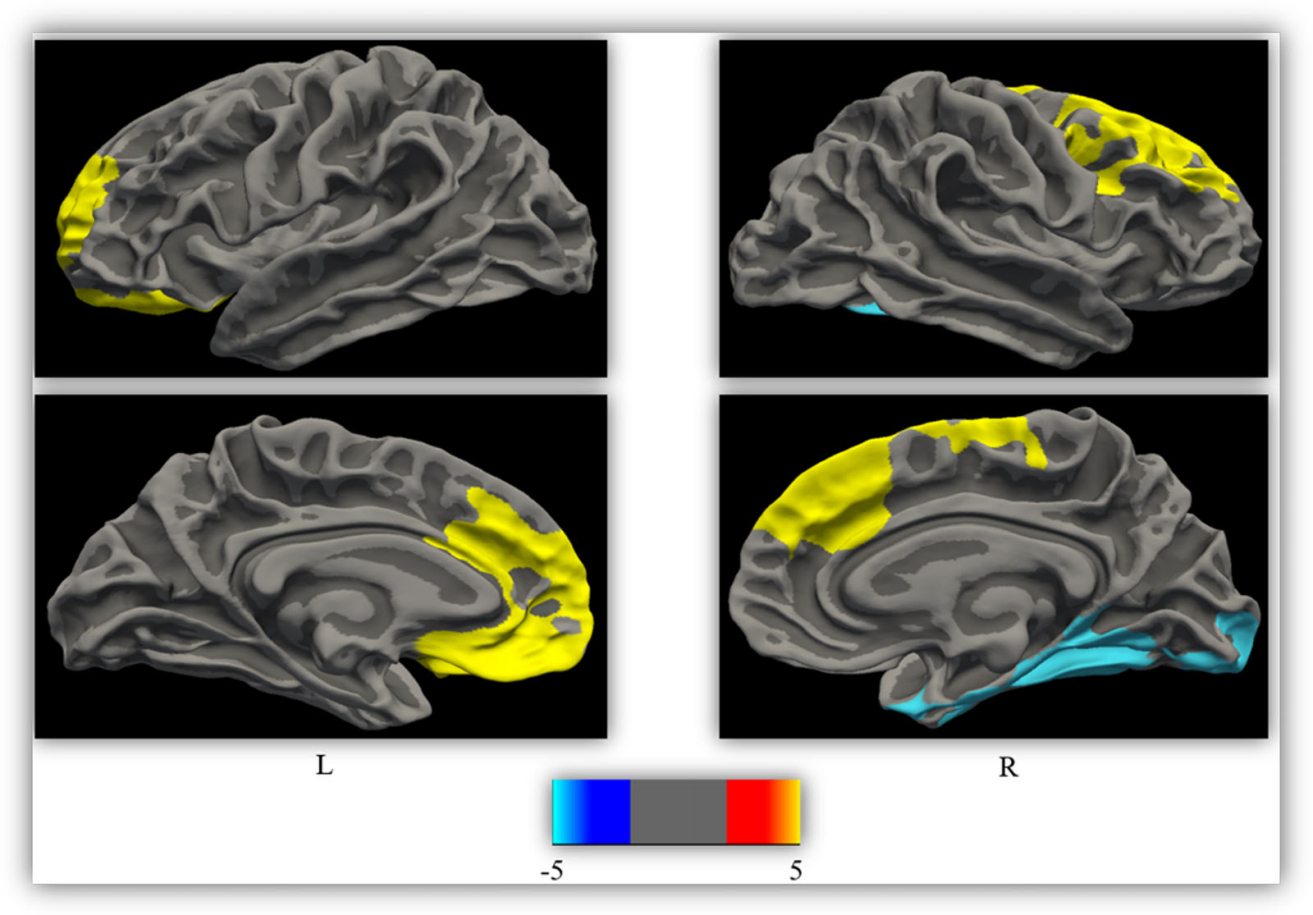
Reduction of local gyrification index (LGI) in patients with iRBD versus HC regions where reduction of LGI were found in patients with iRBD included right rostral middle frontal, right fusiform, and left lateral orbitofrontal. The results of between‐group comparison were performed using a vertex‐wise analysis (Bonferroni‐corrected with Monte Carlo simulation using a *p* value set at < 0.05). The displayed color bar is in the logarithmic scale of *p* values (−log10 [*p* value]). HC, healthy control; iRBD, idiopathic rapid eye movement sleep behavior disorder; L, left hemisphere; R, right hemisphere.

In various brain regions, piRBD patients demonstrated a notable reduction in LGI compared to the HC. Three clusters with significant differences at a threshold of *p* < 0.05 were identified in this study (refer to Table 2 and Figure 2). These clusters encompassed areas of the right fusiform gyrus, right rostral middle frontal gyrus, and left lateral orbitofrontal cortex.

#### 3.2.3 Cortical and Subcortical Volume Analysis

**FIGURE 3:**
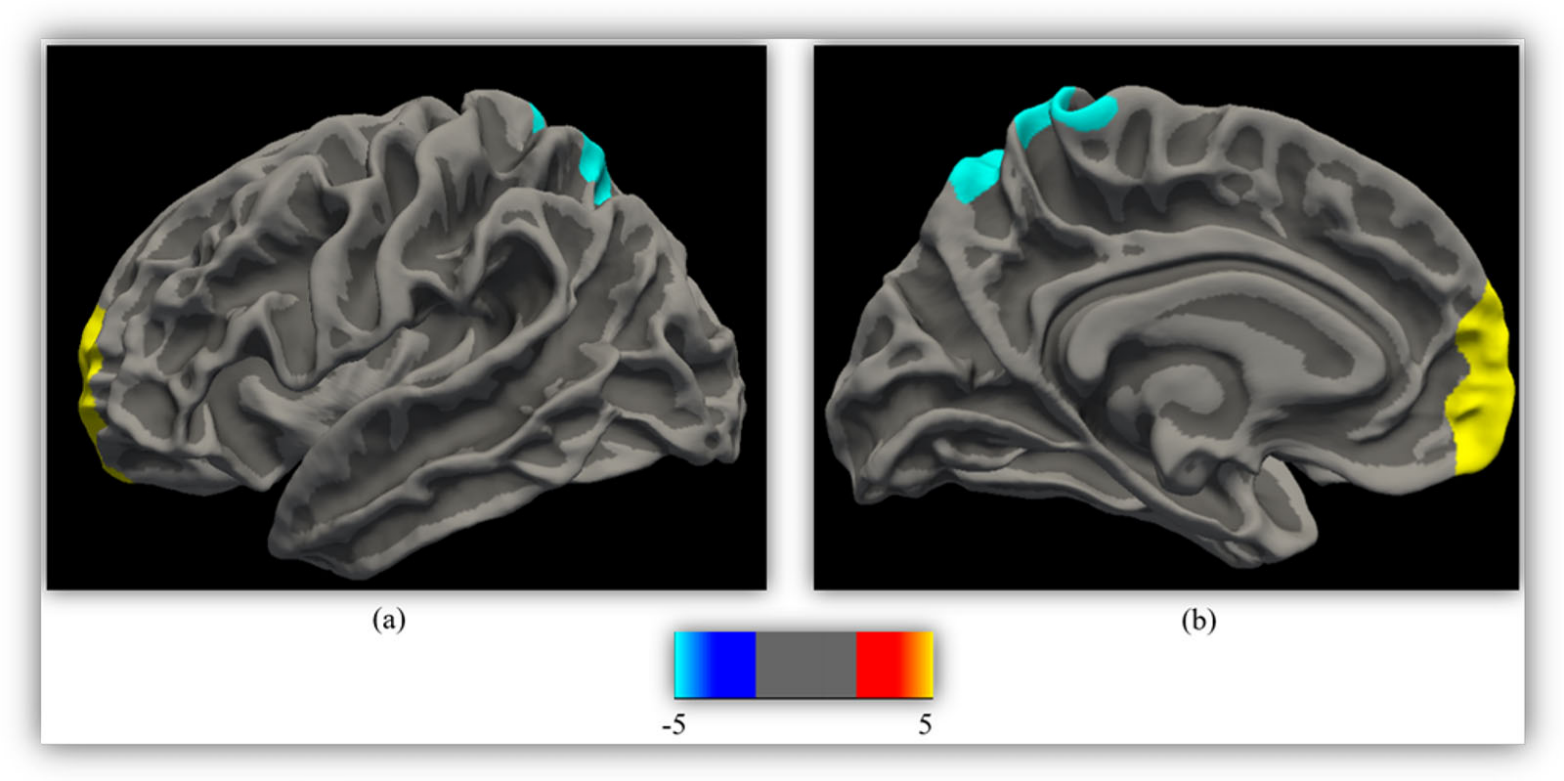
Reduction of cortical volume in patients with iRBD versus HC regions where reductions of cortical volume were found in patients with iRBD included left medial orbitofrontal and left superior parietal. The results of between‐group comparison were performed using a vertex‐wise analysis (Bonferroni‐corrected with Monte Carlo simulation using a *p* value set at < 0.05). The displayed color bar is in the logarithmic scale of *p* values (−log10 [*p* value]): (a) lateral view; (b) medial view. HC, healthy control; iRBD, idiopathic rapid eye movement sleep behavior disorder.

As compared to the HC, a modified GLM revealed significant differences in cortical volume in the left medial orbitofrontal G (Cohen's *d* = −2.67, *p* = 0.005) and left superior parietal G (Cohen's *d* = 2.45, *p* = 0.03) (refer to Table 2 and Figure 3). Additionally, no significant differences were found in subcortical volumes between the two groups.

#### 3.2.4 Sulcus Depth

The modified GLM did not reveal any significant differences in the cortical depth of sulci between the HC and the piRBD groups.

### 3.3 Correlation Analysis

The results of the correlation analyses between SBM and clinical variables are presented in Table 3. Within the piRBD group, a total of eight clusters showed a negative correlation between CTH and the parameters of GDS and LTNS in the frontal, parietal, and temporal regions of the right hemisphere (including the pars orbitalis, pars triangularis, superior parietal, and temporal pole) and the frontal, parietal, and occipital regions of left hemisphere (including rostral middle frontal, precentral, superior parietal, and lateral occipital). Additionally, a significant negative correlation was observed between the volume of cortical regions and GDS in the frontal areas of both the right and left hemispheres (including the right rostral middle frontal and left medial orbitofrontal).

**TABLE 3:** Results of correlation analysis between surface‐based parameters and clinical variables in idiopathic rapid eye movement sleep behavior disorder (iRBD) group.

Parameters	Clinical variable	Cluster size	*N*_vertices_	Coordinate			*p* value	Correlation coefficient	Anatomical region
				*X*	*Y*	*Z*			
Cortical thickness	GDS	5288.64	8855	27.1	−60.8	43.7	< 0.001	−0.35	R superior parietal
		3806.33	5618	40.5	41.5	−5.2	< 0.001	−0.35	R pars orbitalis
		2760.77	5775	37.7	13.4	−37.9	< 0.001	−0.36	R temporal pole
		7655.93	10966	−22.3	−87.6	−10.0	< 0.001	−0.37	L lateral occipital
		1731.66	4021	−14.9	−67.0	54.3	0.002	−0.36	L superior parietal
		1365.82	3293	−53.5	−5.4	36.2	0.015	−0.34	L precentral
Cortical thickness	LTNS	4775.37	7451	46.6	29.9	11.1	< 0.001	−0.36	R pars triangularis
		2087.85	3429	−32.5	30.2	38.5	< 0.001	−0.36	L rostral middle frontal
Cortical volume	GDS	1583.81	2144	19.1	58.1	14.8	< 0.001	−0.35	R rostral middle frontal
		2189.88	3301	−8.3	56.5	−4.5	< 0.001	−0.37	L Medial orbitofrontal

*Note*: L, left; *N*vertices, vertex number at maximum; R, right; *X*Tal, Talairach coordinates for *X* axis; *Y*Tal, Talairach coordinates for *Y* axis; *Z*Tal, Talairach coordinates for *Z* axis.

Abbreviations: GDS, Geriatric Depression Scale; LTNS, letter–number sequencing.

## 4 Discussion

The main aim of this study was to conduct a thorough evaluation of the brain's surface structure, including examinations of CTH, cortical and subcortical volumes, SD, and LGI, as compared to an HC. To our knowledge, the present study is the first to examine the SD and LGI in patients with piRBD.

Compared to the HC, the piRBD group exhibited notable cortical thinning across various brain regions, encompassing the frontal (pars orbitalis, rostral middle frontal G, precentral G), parietal (precuneus G, inferior parietal G, superior parietal G), temporal (temporal pole), and occipital (lateral occipital) regions (Table 2 and Figure 1). These regions are associated with primary motor area, somatosensory association cortex, and secondary visual cortex. The findings of this investigation corroborate earlier research, validating notable decreases in CTH across the frontal, occipital, parietal, and temporal regions, as well as the parieto‐occipital area (Campabadal et al. 2019, 2020; Rahayel et al. 2023; Oltra et al. 2022). Intriguingly, prior studies extensively document cortical irregularities in PD patients, particularly affecting motor and visual regions, which resonate with our observations and imply an implication of these regions in the initial phases of PD (Chaudhary et al. 2021; Zhu et al. 2022). Additionally, our outcomes are in line with structural aberrations noted in past research, particularly in the inferior parietal region among patients with DLB and in the frontotemporal area among those with MSA (Ma et al. 2022; Cao et al. 2021). In the current study, a significant inverse correlation was observed between CTH and GDS and LTNS in the frontal area, especially the opercular gyri and L precentral regions (primary motor cortex) (Table 3). Regarding L precentral region, a previous study found a marginal positive and significant correlation between CTH and RBDSQ in patients with piRBD, although any correlation was not observed between CTH and GDS (Pereira et al. 2019).

Additionally, longitudinal studies have indicated that CTH may serve as a biomarker for phenotypic conversion in patients with piRBD (Campabadal et al. 2020; Pereira et al. 2019). Contrary to the findings of this study, a recent study did not observe any significant differences in CTH between the patient group and the HC (Shin et al. 2023). Variations in results across different studies could be due to the pathological status of study participants, sample size, and methodological factors such as imaging parameters used, image preprocessing methods, artifact correction techniques, and the type of software used for image analysis.

Our findings indicate a significant reduction in the LGI and brain volume in patients, particularly in regions covering the temporo‐occipital lobe (right fusiform), frontal lobe (right rostral middle frontal and left lateral orbitofrontal), and parietal lobe (left superior parietal) (Table 2 and Figure 2). These areas are associated with memory retrieval, executive functions, and the recognition of faces, bodies, words, and numbers. To our knowledge, no study has specifically investigated LGI changes in patients with piRBD, although atrophy in the fusiform, orbitofrontal, left superior parietal, and right rostral middle frontal regions have been reported in past studies (Campabadal et al. 2019, 2020; Donzuso et al. 2023).

The precise mechanism underlying gyrification changes remains unclear. According to van Essen's hypothesis, gyrification changes are primarily caused by tension in the underlying WM connections (both cortico‐cortical and cortico‐subcortical) (Essen 1997). This theory, known as the tension‐based hypothesis, highlights the close relationship between gyrification and WM connectivity (Essen 1997). On the other hand, LGI is a parameter that represents the folding of cortical layers, which develops from the embryonic stage, suggesting its association with neurodevelopmental disorders (White et al. 2010; Chi, Dooling, and Gilles 1977). Findings indicate that there are dynamic changes in cortical folds (degree of gyrification) with age across the lifespan in the cerebral cortex, which can be due to the interaction between early neurodevelopmental and neurodegenerative processes during the course of aging (Garcia et al. 2018; Genç et al. 2023; Gharehgazlou et al. 2022). There is an inversely significant relationship between LGI and age independent of regional surface area on nearly the entire surface of brain (Hogstrom et al. 2013; Schaer et al. 2009; Palaniyappan et al. 2011). This means that LGI decreases with age, which is mostly pronounced in the parietal lobe (postcentral, supramarginal, and inferior parietal lobes) (Hogstrom et al. 2013). LGI also decreases with age non‐linearly in the frontal lobe, indicating this lobe undergoes less change across entire life (Hogstrom et al. 2013). Hence, it can be concluded that changes in the frontal lobe can be more attributed to the neurodegenerative process instead of aging.

It should also be considered that changes in brain gyrification may result from alterations in WM fibers and their connections (Herculano‐Houzel et al. 2010; Klyachko and Stevens 2003; Zhang et al. 2023). In patients with piRBD, WM anomalies have been examined in several studies using diffusion‐weighted imaging (García‐Gomar et al. 2022; Byun et al. 2022; Unger et al. 2010; Alushaj et al. 2024). Remarkably, prior investigations have noted alterations in structural connectivity within the lateral orbitofrontal and rostral middle frontal regions, alongside diminished functional connectivity in frontal areas, such as the left superior frontal gyrus, right precentral gyrus, and right supplementary motor area, among individuals with piRBD in contrast to healthy counterparts (Byun et al. 2022; Unger et al. 2010).

Earlier studies have documented a notable decline in LGI among patients diagnosed with PD, major depressive disorder, multiple sclerosis, Alzheimer's disease, and schizophrenia when compared to control cohorts (Chen et al. 2022; Zhang et al. 2023; Zhang et al. 2021). Extensive literature has highlighted a widespread decrease in gyrification across regions like the middle frontal, superior parietal, and fusiform cortex in PD patients. Zhang et al. demonstrated that a progressive reduction in gyrification is associated with microstructural WM changes, striatal dopamine availability, and serum NfL levels in patients with PD. Alterations of LGI could occur quickly in the early stages of PD because it includes motor cortex region; hence, it could serve as a potential biomarker for monitoring the progression of PD (Tang et al. 2021; Sterling et al. 2016).

Regarding brain volume, several studies using different analysis methods have confirmed volume reduction in the aforementioned regions (Table 3). Using VBM, prior research has noted a decrease in brain volume across various cortical regions, including the frontal, parietal, temporal, and occipital regions, in comparison to control and PD‐pRBD groups (Chen et al. 2022; Donzuso et al. 2023; Woo et al. 2023). Additionally, assessments utilizing [^11^C] donepezil PET tracer, perfusion, and FDG PET have indicated reductions in acetylcholinesterase levels, cerebral blood flow, and glucose metabolism in temporal and frontal cortical areas of piRBD patients relative to controls (Gersel Stokholm et al. 2020; Rahayel et al. 2024; Diaz‐Galvan et al. 2023). Notably, a recent study reported an increase in brain volume in the superior frontal gyrus, possibly indicating compensatory mechanisms in response to early α‐synucleinopathy dysfunction (Donzuso et al. 2023).

However, unlike the present study, no significant variations in subcortical region volumes were observed between the two groups. Recent studies employing VBM analysis have similarly reported no significant differences in subcortical volume between patient groups and healthy individuals (Mala et al. 2024). However, Bourgoin et al. (2019) observed a notable decrease in the volume of the left caudate nucleus and amygdala, extending to the hippocampus, in piRBD patients with depressive and anxiety symptoms compared to the HC. In‐line with this, Campabadal et al. (2019) and Park et al. (2019) reported significant differences in the hippocampal regions and their segments (marked volume reduction) and the thalamus and caudate nucleus (significant increase) in the piRBD group, which contrasts with the current study's findings. Previous studies suggest that, unlike widespread brain volume reductions, increases in brain volume are specific and mainly reported in subcortical areas, such as the thalamus, caudate, and posterior cerebellar lobe (Chen et al. 2022). Several reasons for these discrepancies may include the type of data used, image acquisition methods, data preprocessing techniques, and analysis methods.

No significant differences were found in the SD between the two groups in this study. Similarly, Zhang et al. (2013) demonstrated that the olfactory depth of sulci in piRBD patients does not significantly differ from that of the HC. A widespread reduction in this parameter has been reported in PD. Research has indicated notable variations in SD across different brain regions in PD patients compared to controls. Conversely, another study found increased SD in the parietal region (specifically, the right supramarginal gyrus) in the tremor‐dominant subtype of PD compared to controls, with no significant difference observed compared to the akinetic‐rigid subtype (Li et al. 2022). Additionally, Alzheimer's patients exhibited decreased SD in the parietal lobes compared to controls (Wu et al. 2021). Given that piRBD patients are at a high risk of developing α‐synucleinopathies (73.5% over a 12‐year follow‐up), it is necessary to investigate these brain features in piRBD patients in follow‐up studies. This study revealed structural irregularities in sensory and motor cortical areas (frontal and parietal regions). piRBD is recognized as an early phase of various α‐synucleinopathies such as PD and MSA, all characterized by severe motor dysfunction. Hence, frontal and parietal regions’ atrophy may serve as an early diagnostic indicator for these conditions.

Despite the novel findings of this study, some limitations must be acknowledged. The first limitation is the sample size, which suggests caution in interpreting the results. Therefore, these findings need to be evaluated in a larger cohort group. Second, the RBD diagnosis was made using validated questionnaires but was not confirmed through vPSG. Third, PPMI is a multi‐center cohort study, so naturally, there are apparent differences in MRI scanner and image acquisition methods. To minimize these differences (bias), MRI scans were acquired from the same vendor (3T Siemens scanner). However, inter‐scanner variability should be considered as a possible bias. Fourth, the diagnosis piRBD patients was based on RBDSQ having a sensitivity of 0.96 and a specificity of 0.56. Therefore, the condition was described as piRBD instead of iRBD alone. In spite of this limitation, its use has been recommended by the previous studies (Stiasny‐Kolster et al. 2007; Berardelli et al. 2013).

## 5 Conclusion

Employing comprehensive SBM methods, this investigation analyzed CTH, cortical and subcortical volume, LGI, and SD in individuals diagnosed with iRBD in contrast to a control cohort. The results indicate diminished CTH, volume, and LGI in the frontal and parietal regions, crucial for the cerebral cortex's motor and sensory functions. These alterations hold promise as pivotal indicators for the early identification of the disorder.

## Author Contributions

**Milad Najafzadeh**: conceptualization, methodology, software, investigation, visualization, writing–original draft, writing–review and editing. **Athareh Saeeidian‐Mehr**: methodology, validation. **Hossein Akbari‐Lalimi**: conceptualization, methodology, validation. **Zohre Ganji**: methodology, validation. **Shahrokh Nasseri**: conceptualization, supervision, formal analysis, software, validation, writing–review and editing, funding acquisition, project administration. **Hoda Zare**: conceptualization, methodology, validation, software, formal analysis, supervision, writing–review and editing. **Luigi Ferini‐Strambi**: writing–review and editing.

## Ethics Statement

The data utilized in this article were taken from the PPMI database. All participating PPMI sites received approval from an ethical standards committee prior to study initiation; for a list of participant sites, see https://www.ppmi‐info.org/about‐ppmi/ppmi‐clinical‐sites. Central IRB approval provided by CWG IRB (tracking number: 20200597; current clinical trial identifier of PPMI study in clinical trials.gov: NCT04477785). The authors confirm that the ethical policies of the journal, as noted on the journal's author guidelines page, have been adhered to and the appropriate ethical review committee approval has been received. The US National Research Council's guidelines for the Care and Use of Laboratory Animals were followed.

## Consent

All participants provided written informed consent.

## Conflicts of Interest

The authors declare no conflicts of interest.

### Peer Review

The peer review history for this article is available at https://publons.com/publon/10.1002/brb3.70057.

## Data Availability

Data used in the preparation of this article were obtained (on January 10, 2024) from the Parkinson's Progression Markers Initiative (PPMI) database (www.ppmi‐info.org/access‐data‐specimens/download‐data), RRID:SCR_006431. For up‐to‐date information on the study, visit www.ppmi‐info.org.
